# Coping with COVID-19: Survey data assessing psychological distress to COVID-19 and vaccine hesitancy with measures of theory of planned behavior, mindfulness, compassion, cultural orientation, and pandemic fatigue

**DOI:** 10.1016/j.dib.2022.108390

**Published:** 2022-06-14

**Authors:** Emily A. Mueller, Piraorn Suvanbenjakule, Chung Xiann Lim, William H. O'Brien, Jennifer Chavanovanich, Somboon Jarukasemthawee, Kullaya Pisitsungkagarn, Panita Suavansri

**Affiliations:** a Bowling Green State University, United States; b Chulalongkorn University, Thailand

**Keywords:** Intolerance of uncertainty, Authoritarianism, Psychological flexibility, Perceived susceptibility to COVID-19

## Abstract

As the COVID-19 pandemic extends into another year, the causes and consequences of pandemic fatigue and vaccine hesitancy have become prominent concerns. This dataset contains MTurk survey responses from 658 vaccinated USA samples indicating: (a) pandemic fatigue and psychological distress (physical and trauma symptoms); (b) delays in receiving medical care due to COVID-19 restrictions; (c) vaccine-related behavior and beliefs (type of vaccine and vaccine hesitancy), and (d) COVID-19 preventive health behaviors. Several predictor variables were also collected including: (a) demographic variables; (b) COVID-19 health risk factors; (c) perceived susceptibility to disease and intolerance of uncertainty; (d) attitudes, subjective norms and perceived behavioral control about COVID-19 vaccine from the theory of planned behavior; (e) compassion for self and others; (f) psychological flexibility and inflexibility; (g) Buddhist mindfulness insight (impermanence, acceptance of suffering, nonself attachment, mindfulness); and (h) cultural orientation and authoritarianism. The data were collected between August 28th and October 18th of 2021. Out of the 746 MTurk workers who responded to the survey, 88 were removed from the dataset due to failing attention checks and problems with quality data. The responses from the remaining 658 allow an examination of the associations between fatigue and distress from COVID-19; COVID-19 vaccine related behaviors and beliefs; preventive health behaviors for COVID-19; COVID-19 susceptibility; intolerance of uncertainty; together with compassion, psychological flexibility, mindfulness, cultural orientation, as well as authoritarianism as possible moderators of COVID-19 fatigue, distress, and vaccine beliefs.

## Specifications Table

**Table:** 

Subject	Psychiatry and Mental Health
Specific subject area	Measurement of COVID-19 fatigue and distress, objective risk factors, perceived risk, attitudes and beliefs about COVID-19 vaccines, compassion, mindfulness, psychological flexibility, cultural orientation, authoritarianism.
Type of data	Tables. Scale summary (subscale and total scores) and demographic data.
How data were acquired	The survey containing all related measures was published online on MTurk. A copy of the survey can be obtained from the corresponding author.
Data format	Filtered
Description of data collection	The announcements detailing the survey were published on Amazon Mechanical Turk on August 28th and October 5th. The participants were given a consent form through a link. With the consent form signed, the participants were redirected to a survey on Qualtrics. Participants were compensated for $1.50. The survey link was open until October 18th, 2021. After data were cleaned, 658 participant responses were kept in the dataset. The survey items can be provided by the corresponding author. Fig. 1 depicts the participant's geographical location. Tables 1 and 2 provide descriptive statistics and basic psychometric properties of the measures.
Data source location	Data source location: William H. O'Brien, Ph.D. Department of Psychology, Bowling Green State University, Bowling Green, Ohio 43402, USA.
Data accessibility	Repository name: Harvard Dataverse Direct URL to data: https://doi.org/10.7910/DVN/JTVZF2

**Value of the Data**

• Data provide information on pandemic fatigue, vaccine hesitancy, and COVID-19 related distress. Predictors may include demographic characteristics, perceived susceptibility to COVID, Perceived Vulnerability To Disease (PVD), compassion for others (CS), self-compassion (SCS), authoritarianism (VSA), mindful insight (MIS), Theory Of Planned Behavior (TPB), psychological flexibility and inflexibility (MPFI), intolerance of uncertainty (IUS), pandemic fatigue (PFS), vaccine hesitancy (VHS), social distancing, physical stress-related symptoms (PHQ-15), trauma symptoms (IES-R), and individualism/collectivism.
• Data can be used for structural equation modeling and testing of preventive health behaviors and psychological coping with the COVID-19 pandemic.
• Data can also be used to evaluate the ways that theory of planned behavior, mindfulness, and cultural orientation variables are related to pandemic fatigue and vaccine hesitancy which can be used to inform COVID-19 health promotion interventions.

## 1 Data Description

**Fig. 1:**
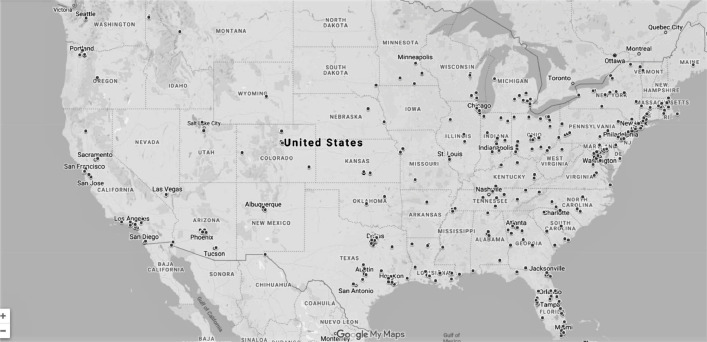
Geographic distribution of time 1 dataset (retrieved from Google my maps).

The data set is available in SAV format [1]. Fig. 1 depicts the geographical location of participants in the dataset. The provided descriptive statistics as well as basic psychometric properties of the measures are shown in Tables 1 and 2. Supplementary material contains the informed consent, instructions, and full questionnaire that was provided to participants during data collection.

**Table 1:** Constructs, measure description, and statistics.

Construct	Measure	# of Items	Cronbach's Alpha	Mean (SD)	Skew (SE = .10)	Kurtosis (SE = .20)
Dependent Variables						

Vaccine Hesitancy	Confidence subscale	3	.584	4.05 (.61)	-.75	1.12
	Complacency subscale	3	.826	3.29 (1.11)	-.73	-.55
	Constraints subscale	3	.819	3.35 (1.07)	-.88	-.23
	Calculation subscale	3	.571	3.85 (.68)	-.88	1.82
	Collective responsibility subscale	3	.281	3.53 (.76)	.74	-.17
COVID-19 Pandemic Fatigue	Pandemic Fatigue Scale – Behavioral fatigue subscale	7	.916	4.78 (1.33)	-1.03	.61
	Pandemic Fatigue Scale – Information fatigue	3	.767	5.01 (1.31)	-1.01	.71
Post-Trauma Stress	Impact of Events Scale	22	.970	2.25 (.97)	-.87	.09
Physical Symptoms	Patient Health Questionnaire-15	14	.898	.83 (.49)	-.06	1.06
Quality of Life	Selected items from the WHO Quality of Life Scale	5	.771	.79 (.12)	-.71	.94
COVID-19 Post Traumatic Growth	Post Traumatic Growth Inventory	10	.917	3.37 (.96)	-1.38	2.34
COVID-19 Medical Care Delays	Created items	4	.784	3.49 (.88)	-1.08	.84
Mental Health Symptoms	General Health Questionnaire	12	N/A	N/A	N/A	N/A
	Positively worded	6	.856	3.33 (81)	-.68	.36
	Negatively worded	6	.811	2.43 (.67)	.34	-.01
COVID-19 Health Protective Behaviors – Avoiding Public Settings and Contact with People	Preventative Actions Taken Scale	2	N/A	3.01 (.69)	-.53	-.06
COVID-19 Health Protective Behaviors – PPE USe	Preventive Actions Taken Scale	2	N/A	3.04 (.67)	-.58	.15

Predictor Variables						

COVID-19 Vaccination Attitudes, Subjective Norms, Perceived Behavioral Control	Theory of Planned Behavior Scale created items – Attitude	6	.749	3.42 (.76)	.74	-.17
	Theory of Planned Behavior Scale created items – Subjective norms	3	.557	4.04 (.61)	-.61	.79
	Theory of Planned Behavior Scale created items – Perceived behavioral control	5	.552	3.50 (.62)	.92	.53
Perceived Efficacy of COVID-19 Vaccines	Created Items	2	N/A	3.98 (.70)	-.67	.78
Intolerance of Uncertainty	Intolerance of Uncertainty Inventory	12	.902	3.50 (.75)	-.82	.55
Perceived Vulnerability to COVID-19(?)	Single Item	1	N/A	3.28 (.96)	-.44	.48
General Perceived Vulnerability to Disease	Perceived Vulnerability to Disease Scale	4	.848	3.42 (.93)	-.92	.20
Compassion	The Compassion Scale	14	.727	3.56 (.47)	.48	.77
Self-Compassion	Self-Compassion Scale	12	.630	3.08 (.47)	.58	4.42
Serenity	Serenity Scale	22	.923	3.71 (57)	-.63	1.56
Psychological Flexibility	Multidimensional Psychological Flexibility Inventory- Psychological Flexibility	12	.889	4.37 (.75)	-.34	.03
	Multidimensional Psychological Flexibility Inventory- Psychological Inflexibility	12	.939	3.94 (1.10)	-.70	-.10
Mindfulness Insight	Mindful Insight Scale	47	.806	4.62 (.42)	.17	2.38
Individualism and Collectivism	Cultural Orientation Scale– Individualism subscale	8	.738	3.20 (.74)	-.32	.06
	Cultural Orientation Scale– Collectivism subscale	8	.786	3.27 (.76)	-.17	.01
Authoritarianism	Very Short Authoritarianism Scale	6	.476	3.02 (.60)	-.90	2.96

On 10 Dec BE 2564, at 05:16, WHOB <williamobrien@gmail.com> wrote:

who completed thin under 700 s. I also attached the description of measures. I still owe you a description of the compassion scale.

**Table 2:** Demographic characteristics of the sample.

	*N* = 658		
Variable	M	SD	%
Age	37.22	11.18	
Gender			
Female			47
Male			53
Marital Status			
Single			13
Married			82
Cohabitating			02
In Long Term Relationship, Not			01
Cohabitating			
Divorced			1
Widowed			1
Employment Status			
Employed 1–23 h/week			15
Employed 24–39 h/week			30
Employed > 40 h/week			53
Not employed/looking			1
Not employed/not looking			1
Retired			1
Disabled			1
Race/Ethnicity^1^			
Hispanic/Latinx			4
White			37
Black/African			5
American			1
Asian			1
Pacific Islander			1
American Indian or Alaska Native			1
Two or more			1
Unidentified			52*
Educational Attainment^1^			
High School			2
Some College			4
Associates Degree			1
Bachelors Degree			34
Masters Degree			10
Beyond Masters			1
Missing			48
Have Children? (Yes)			81
Number of Children	1.40	.91	
Annual Income in Dollars			
<20,000			9
20,000–40,000			18
40,000–60,000			36
60,000–80,000			11
80,000–100, 000			10
100,000–120,000			4
120,000 –140,000			2
>140,000			3
NonCOVID-19 Illness (yes)			8
Taking Medication (yes)			6

Note:

⁎ Race and education were not recorded in the first data collection date due to a technical error. Therefore, there are significant missing data on these two variables.

## 2 Experimental Design, Materials and Methods

A team of researchers from Chulalongkorn University, Bangkok, Thailand, and Bowling Green State University, Bowling Green, Ohio, USA put together the survey. The project received an approval from the Bowling Green State University Institutional Review Board (#1562479-4). Initially, a total of 746 Amazon Mechanical Turk Workers with high rating participated through CloudResearch from August 28th to October 18th 2021. If any of these issues were discovered, the participant's responses were deleted: (a) less than 75% completion rate, (b) duplicated IP address, (c) failing more than 1 of 5 attention check items, (d) improbable or nonsense responses to open ended questions about height and weight, or (e) an extremely short or long amount of time to finish. The final number of participants was 658.

The variables and scale descriptions are detailed in Table 1. Table 2 provided the summarized demographic characteristics of the participants together with their geographic distribution presented in Fig. 1.

There are concerns and questions about the representativeness of MTurk samples. This sample is typically as representative of the US population as undergraduate student samples, samples gathered in university neighborhoods, and convenience samples do, but less so than national probability samples [2]. Nonetheless, researchers [2] pointed out that there is a potential bias in national probability samples due to over sampling with older adults and individuals with higher conservatism.

MTurk samples could demonstrate a level of strength. The only appropriate approach to obtain survey data across different populations and regions is through an online channel while COVID-19 regulations are in place. We proceeded to screened for response quality with caution by utilizing a list of techniques, such as captchas, attention checks, open-ended questions, and analyses of unusual response times [3].

## 3 Measures

### 3.1 Demographics

Basic demographic items asked the participants to specify their age, gender, religion, marital status, ethnicity, education, children, income, housing conditions, and work conditions and differences caused by COVID-19. They indicated their current illnesses and medication taken.

### 3.2 Vaccine Type and Vaccine Hesitancy

Participants reported when they received the vaccine and which vaccine they received. Participants then completed a 15-item Vaccine Hesitancy Scale (VHS) that assessed the degree to which barriers to obtaining a vaccine influenced their decision. The construct is divided into five subscales: confidence (in the vaccine), complacency, constraints, calculation, and collective responsibility. Responses range from “Strongly Disagree” (1) to “Strongly Agree” (5) on a Likert scale. A sample item from the constraint subscale is, “For me, it was inconvenient to receive the COVID-19 vaccine.” The subscales had poor to good internal consistency, ranging from *α* = .28 to .83. The complacency and constraints subscales had Cronbach's alphas above .80, while the collective responsibility subscale had a Cronbach's alpha of .28.

### 3.3 COVID-19 Related Interference/Delays in Receiving Medical Care

Four items were created to evaluate the extent to which participants were unable to receive timely medical care due to COVID-19 restrictions. The four items were: “In order to receive medical care during the COVID-19 pandemic, I had to choose between the risk of becoming infected with COVID-19 and the risk of my symptoms getting worse,” “I am worried that if I cannot get enough medical treatment during COVID-19, my illnesses will get worse,” “As a result of the COVID-19 outbreak, I have lost the opportunity to be as healthy as I would like to be,” “I have a health problem for which I was unable to receive medical treatment because of COVID-19.” Responses range from “Strongly Disagree” (1) to “Strongly Agree” (5) on a Likert scale. More COVID-19-related medical care interference is signified by higher scores.

### 3.4 COVID-19 Preventive Actions Taken Scale

Research findings up to that point provided the foundation for the Preventative Actions Taken Scale (PATS) development in late January, 2020 [4]. The initial 12-item assessed participants in China and the United States how likely they were to engage in COVID-19 prevention practices. Responses range from “Does not apply to me at all” (1) to “Applies to me very much or most of the time” (4). Psychometric evaluation of the PATs indicated that it had two factors. The first factor with 5 items assessed public settings avoidance and contact with people (e.g., “I avoid public events and crowded places”). The second factor with 3 items assessed PPE use (“I wear a mask everywhere”). Both of the subscales provided good internal consistencies showing Cronbach's alpha level at .76 and .77, respectively. We selected the two most highly correlated items from each factor (for a total of four items) to reduce participant burden and redundancy in the items. For each factor, a total score was generated. A greater participation in preventative action taken is signified by higher scores.

### 3.5 Pandemic Fatigue Scale

The Pandemic Fatigue Scale (PFS) developed by Lilleholt et al. [5] contains 10 self-report items. The construct of pandemic fatigue was described by the authors as “a general feeling of demotivation towards following COVID-19 related health-protective behaviors and staying informed about the development of the pandemic” (p. 5). Lilleholt et al. [5] constructed and refined the measure using a large sample of participants and identified two factors: (a) information fatigue (e.g., “I am tired of all the COVID-19 discussions in TV shows, newspapers, and radio programs, etc.”) and (b) behavioral fatigue (e.g., “I am tired of restricting my liberty to avoid the spread of COVID-19”). Responses range from “Strongly Disagree” (1) to “Strongly Agree” (7) on a Likert scale. Higher levels of fatigue is signified by higher scores.

### 3.6 Trauma Symptoms

The Impact of Events Scale – Revised (IES-R) assessed trauma-related psychological symptoms and has been extensively used as a measure of post-traumatic stress symptoms in community and clinical samples [6]. The scale comprised of 22 items (e.g., “I thought about it when I didn't mean to”). Responses range from “not at all” (0) to “extremely” (4) on a Likert scale. The instructions stated: “The following is a list of difficulties people sometimes have after stressful life events. Please read each item, and then indicate how distressing each difficulty has been for you during the past 7 days with respect to the Coronavirus situation.” The calculation for the IES-R total score was carried out. Greater trauma symptoms are signified by higher scores.

COVID-19 post-traumatic growth was measured using the short form of the post-traumatic growth inventory (PTGI-SF) [7]. The scale consists of 10 items concerning one's sense of spirituality, religion, closeness with others, and life path (e.g., “I established a new path for my life” and “I have a better understanding of spiritual matters.”). Participants were instructed to indicate if any of the described changes occurred in their lives as a result of the COVID-19 pandemic, ranging from “I did not experience this change” (0) to “I experienced this change to a very great degree (5). A total PTG score was calculated by taking the sum of all 10 items, where a higher score indicates a greater degree of post-traumatic growth.

### 3.7 Physical Symptoms

The patient health questionnaire-15 [8] measured stress-related physical symptoms. An item concerning menstrual symptoms was excluded as it did not apply to all participants. Other items (e.g., “headaches”) were on a 3-point Likert scale ranging from “not bothered at all” (0) to “bothered a lot”. The assessment indicated the level each symptom bothered them in the past month. (2). Researchers [9] reported that PHQ-15 has acceptable validity and internal consistency. Calculation for a total score was carried out. More physical symptoms are signified by higher scores.

### 3.8 Mental Health

The short general health questionnaire (GHQ 12) was used to assess participants’ psychological distress [10]. This scale was developed by Goldberg in 1972 to detect nonpsychotic psychological impairment over the past month and requires respondents to rate 12 items (e.g., “lost sleep over worry” and “felt that you couldn't overcome your difficulties”) [10]. Items were rated from “much less than usual” (0) to “much more than usual” (4). A total GHQ was calculated by summing the 12 items (reverse coding positive items 1, 3, 4, 7, 8, and 12). A higher score indicates more psychological distress over the last month. The validity of the GHQ 12 has been found to be acceptable when using the Likert method for scoring (*α* = .73) [11]. The validity in the current study was lower at .52.

### 3.9 Perceived Susceptibility to COVID-19

The researchers created a single item to measure perceived vulnerability to COVID-19. The question asked, “How likely is it that you will contract COVID-19?” Responses included “no chance” to “certain” on a 5-point scale. In the preventive behavior literature, using one perceived susceptibility item has been supported [12]. The single-item measure of perceived susceptibility was used as an index of absolute risk because complex and multi-item perceived susceptibility items have been found to have problematic features, such as confounding (e.g., containing items that assess other constructs such as perceived severity or self -comparison with others) [29]. Additionally, single item measures of unidimensional constructs have the advantage of avoiding redundancy in a scale and reducing participant fatigue.

### 3.10 Perceived Vulnerability to Disease

The perceived vulnerability to disease scale (PVD) was developed to assess overall perceived risk for illness with 15 items [13]. The responses were ranging from “Strongly disagree” (1) to “Strongly agree” (5) for each item (e.g., "My past experiences make me believe I am not likely to get sick even when my friends are sick." Our prior examination of the psychometric properties of the PVD revealed suboptimal Cronbach's alphas [4]. An exploratory factor analysis with an oblimin rotation was conducted to determine subscales that match the present sample. According to the results, the three-factor model fits data well: (a) general perceived vulnerability to disease (e.g., “In general, I am very susceptible to colds, flu, and other infectious diseases''), (b) perceived immunity (e.g., “my immune system protects me from most illnesses that other people get” – these items were reversed coded) and (c) germ aversion (e.g., “I do not like to write with a pencil someone else has obviously chewed on”). For this data set, only the 4 non-reversed perceived vulnerability items were used. A higher sense of vulnerability is signified by higher scores.

### 3.11 Intolerance of Uncertainty

The Intolerance of Uncertainty Scale (IUS) contains 12 items [14] assessing the psychological distress related to ambiguous and unpredictable situations (e.g., “It frustrates me not having all the information I need”). Responses were on a 5-point scale ranging from “Not at all characteristic of me” (1) to “Entirely characteristic of me” (5). The student and community samples were recruited to test the IUS measure [15]. The results show that it could reflect inhibitory and prospective anxiety. The calculation for the IUS total score was carried out. Greater intolerance of uncertainty is signified by higher scores.

### 3.12 Quality of Life

The survey included 5 out of 26 items of World Health Organization Quality of Life Brief scale [16]. Responses had different 5-point scale labels, such as “never” to “very often” and “very dissatisfied” to “very satisfied.” The 5 items assessed: overall life satisfaction, health satisfaction, work satisfaction, enjoyment of life, and life meaningfulness. The calculation for a total score for these 5 items was carried out. Better perceived quality of life is signified by higher scores.

### 3.13 Self-Compassion and Other Compassion

The Self-Compassion Scale (SCS) contains 26 self-report items developed by Neff [17] to measure kindness versus judgment, common humanity versus isolation, and mindfulness versus overidentification. It has been used in many research investigations with reports of acceptable psychometric properties including internal consistency, test-retest reliability, and validity [18]. The SCS yields 6 subscales: Self-Kindness, Common Humanity, Mindfulness, Self-Judgment, Isolation, and Over-Identified. In this data set the total SCS score and 6 subscale scores are provided. Greater compassion is signified by higher scores.

### 3.14 The Compassion Scale

The Compassion Scale (CS) contains 14 items created to assess how much compassion one has towards others [19]. The scale is divided into six subscales: Kindness, Common Humanity, Mindfulness, Indifference, Separation, and Disengagement. All items are summed to create a composite score. Responses range from “Almost Never” (1) to “Almost Always” (5) on a Likert scale. A sample item is, “I like to be there for others in times of difficulty.” The reliability of this measure has been found to be good, ranging from .78 to .90 [19]. The composite Mindfulness score using the current sample showed good reliability, with a Cronbach's alpha of .73.

### 3.15 Psychological Flexibility and Inflexibility

The Multidimensional Psychological Flexibility Inventory-24 (MPFI-24) [20] assesses psychological flexibility (e.g. “I tried to make peace with my negative thoughts and feelings rather than resisting them”) and inflexibility (e.g. “I thought some of my emotions were bad or inappropriate and I should not feel them”) with 24 items. Responses range from “Never true” (1) to “Always true” (6) on a 6-point Likert scale. Six subscales of Psychological Flexibility (Acceptance, Present Moment Awareness, Self-as context, Defusion, Values, Committed Action) and six subscales of Psychological Inflexibility (Experiential Avoidance, Lack of contact with the present moment, Self-as content, Fusion, Lack of contact with values, Inaction) corresponding to the Hexaflex Model were yielded [21]. Higher scores indicate higher degree of the domain measures. Global average scores for psychological flexibility and psychological inflexibility were derived by averaging their corresponding subscales. Both global composite scores displayed good internal consistency (Psychological Flexibility, *α* = 0.89; Psychological Inflexibility *α* = 0.94) for the present sample.

### 3.16 Insight Scale

The Mindful Insight Scale (MIS) measures Buddhist insight into the three characteristics of existence (suffering, impermanence, and non-self attachment) with 47 items [22]. Responses range from “very untrue of my experience” (1) to “very true of my experience” (7) on a Likert scale. Sample items from each of the three categories are: “I calmly accept physical suffering is a part of being human,” “I am aware and calmly accept that my feelings and emotions change constantly,” and “I am aware that I am just a small element of this great universe.” It has acquired good internal consistency and criterion validity from the prior work [22]. The scale displayed acceptable level of reliability with Cronbach's alpha level at .81. Higher scores on the MIS indicate greater mindful insight.

### 3.17 Individualism and Collectivism

Individualistic and collectivistic orientation were measured with Self-Construal scale having 30 items in total [23,24]. Responses range from “Strongly disagree” (1) to “Strongly agree” (7) on a Likert scale. Half of the items made up the independence/individualism subscale and the other half made up the interdependence/collectivism subscale. Steel et al. [24] demonstrated the theoretical equivalence between the constructs of independence and interdependence, and individualism and collectivism. Example items assessing individualism and collectivism are “I enjoy being unique and different from others in many respects” and “Even when I strongly disagree with group members, I avoid an argument”, respectively. Greater scores suggest higher levels of individualism/collectivism. The Cronbach's alphas of both subscales were satisfactory (*α* = .74 and .79) in the present sample.

### 3.18 Serenity Scale

The 22-item brief serenity scale was used to assess participants’ serenity and sense of inner peace, with subscales measuring Inner Haven, Trust, Acceptance, Perspective, Benevolence, and Precent-Centeredness [25]. A total Serenity construct was calculated by taking the sum of all 22 items, where a higher score indicated more serenity. Respondents were asked to endorse items such as “I accept situations that I cannot change,” and “I feel serene.” Responses ranged from “Never” (1) to “Always” (5). The Total Serenity construct has been found internally consistent and reliable with a Cronbach's alpha of .93 [25]. The reliability in the current study was also high with a Cronbach's alpha of .92.

### 3.19 Very Short Authoritarianism Scale

The very short authoritarianism scale (VSA) was used to assess authoritarianism, conservatism, and traditionalism in participants with 6 items [26]. Responses range from “Strongly Disagree” (1) to “Strongly Agree” (5) on a Likert scale. For example, “What our country needs most is discipline, with everyone following our leaders in unity.” The reliability for the VSA was acceptable in a previous work, with a Cronbach's alpha of .73 [26]. The reliability in the current study was lower, with a Cronbach's alpha of .48. A higher score on the VSA indicates higher authoritarianistic, conservative, and traditionalist tendencies.

### 3.20 Theory of Planned Behavior

The scales measuring the theory of planned behavior predictors were used to assess attitude towards the vaccine, subjective norms, and perceived behavioral control. First, the attitude measure was adapted from Drążkowski and Trepanowski [27]. The positively worded item is such as, “Getting vaccinated for COVID-19 seems to me to be very wise.” The negatively worded item is such as: “Getting vaccinated for COVID-19 seems to me to be very harmful.” A higher score after reversing the negatively phrased items will reflect greater positive attitude towards the COVID-19 vaccine. Second, the subjective norms measure by Chu and Liu [28] assesses an individual's perception of descriptive and injunctive norms about getting a COVID-19 vaccine with the composite reliability at .66. The descriptive norms subscale items describe what the people who are like them or important to them would do. An example of the item is: “Most people who are like me will get vaccinated for COVID-19.” Similarly, an injunctive norms subscale item refers to what people think others think they should do. The item states, “Most people who are important to me think that I should get COVID-19 vaccines.” A higher score will reflect more subjective norms. Lastly, for perceived behavioral control, the behavioral control scale [27] was adapted to assess an individual's belief on how much control they think they have in order to get a COVID-19 vaccine (*α* = .75). A sample item is, “If I wanted to, I could go to a medical facility with no problems whatsoever and get vaccinated, when that becomes possible” and “Getting vaccinated against COVID-19 would be very easy for me.” A higher score reflects more control the person believes they have over getting a COVID-19 vaccine. Responses range from “strongly disagree” (1) to “strongly agree” (5) on a Likert scale.

## 4 Procedure

The researchers announced the study survey on Amazon Mechanical Turk on August 28th and October 5th, 2021. An informed consent was present to each participant. If they provide their agreement to participate, they were then redirected to the questionnaire. There were 5 attention check items and 3 captcha items throughout the survey. The participants were asked if they intended to skip the item(s) if they skipped an item before getting to the next page. If the participant selected “yes”, showing their intention to skip the item, they would continue to the next page. If they selected “no”, the skipped item was presented again. Participants were compensated for $1.50 for finishing the questionnaire.

## Ethics Statement

The project received an approval from the Bowling Green State University Institutional Review Board. It also followed all of the APA ethical guidelines. Before each participant gave their responses, they were given the opportunity to provide their informed consent. No stress-inducing stimuli was present throughout the survey and there was no foreseen risk associated with participation. Participants may stop at any moment with no repercussions. All participant's identities could not be discovered through nor linked with the data. Hence, participant responses were completely anonymous. The IRB number for this project is 1562479-7

## CRediT authorship contribution statement

**Emily A. Mueller:** Conceptualization, Methodology, Data curation, Project administration, Writing – original draft, Writing – review & editing, Visualization. **Piraorn Suvanbenjakule:** Conceptualization, Methodology, Data curation, Writing – original draft, Writing – review & editing, Visualization. **Chung Xiann Lim:** Conceptualization, Methodology, Investigation, Writing – original draft, Writing – review & editing, Visualization. **William H. O'Brien:** Supervision, Project administration, Funding acquisition, Conceptualization, Methodology, Investigation, Writing – original draft. **Jennifer Chavanovanich:** Conceptualization, Methodology, Resources, Writing – review & editing. **Somboon Jarukasemthawee:** Conceptualization, Methodology, Resources, Writing – review & editing. **Kullaya Pisitsungkagarn:** Conceptualization, Methodology, Resources, Writing – review & editing. **Panita Suavansri:** Conceptualization, Methodology, Resources, Writing – review & editing.

## Declaration of Competing Interest

All authors declare that they have no known competing financial interests or personal relationships which have, or could be perceived to have, influenced the work reported in this article.

## Data Availability

Vaccine Hesitancy *n* = 658 SPSS.sav (Original data) (Dataverse).

## Appendix Supplementary materials

Image, application 1
