# Supplementary material for: Coping with COVID-19: Survey data assessing psychological distress to COVID-19 and vaccine hesitancy with measures of theory of planned behavior, mindfulness, compassion, cultural orientation, and pandemic fatigue
Source: Data Brief. 2022 Jun 14;43:108390. doi: 10.1016/j.dib.2022.108390 (PMC9195342; doi:10.1016/j.dib.2022.108390)
Supplement: Supplementary file 1 [file mmc1.docx]

OMapLab COVID-19 (US) - MTURK - New survey July 2021

Start of Block: Informed Consent

Informed Consent
 Description of the Study: 
 You are invited to participate a research study by filling out a survey now and a follow-up survey in 3 months. You will be paid $1.50 for each completed survey on MTurk. This study is designed to understand how people have been experiencing the COVID-19 health situation.  This research study is being conducted by Dr. William H. O’Brien. Dr. William O’Brien is a professor of psychology at Bowling Green State University, USA. You must be at least 18 years old to participate in the study.
 Summary of Involvement: 
 If you agree to participate, you will be invited to complete the online survey that follows this consent form. In the survey, you will be asked to answer questions about your experiences and reactions to the COVID-19 situation. The survey will take about 30 minutes to complete. You may skip any question that do not want to answer.  You can put a 4-digit number on your survey that you can remember.  In three months we will you  a link for an follow-up survey. If you choose to do that survey, you will put your personal 4-digit number on it so we can match the surveys.  We will not know your name, your email, or have any way to link your responses to any person. That way all surveys are anonymous.   
 Confidentiality:  
 If you agree to participate, your survey answers will be anonymous. This means that no one will be able to link your name to your survey responses. Your identity will not be known by us or anyone else.           
 Risks, Benefits, and Voluntary Status:
 There are no foreseeable risks associated with this study, meaning that by participating in this study you will not encounter any more risk than you encounter in your regular workday. You may skip any questions that you do not want to answer, and you may stop participating at any time. You do not have to complete the follow-up survey.
 You will be paid $1.50 through MTurk for completing each survey. The main social benefits of this research is that it will help us:  (a) learn about how people are experiencing the COVID-19 situation, (b) the different things that people do to cope with the COVID--19 situation, and (c) ways that we can help people who may be having a hard time coping.

 Your participation in this study is voluntary. You are free to withdraw consent and end participation in the study at any time without penalty. You have the right to have all questions concerning the study answered by the researcher. You may request a copy of the results of the study. You can also download and save a copy of this consent form if you wish.
 Contact Information: 
 If you have any questions or comments about this study, you may contact William O’Brien, Ph.D. by email ([wobrien@bgsu.edu](mailto:wobrien@bgsu.edu)) or telephone 419-654-1500. If you have any questions regarding the conduct of this study or about your rights as a research participant, you may contact the Chair of Bowling Green State University’s Institutional Review Board (IRB) by phone at 1- 419-372-7716 or by email at [orc@bgsu.edu](mailto:orc@bgsu.edu).

 ---------------------------------------------------------------------------------------------------------------------

 Voluntary Consent:  By clicking on the proceed button below, I agree to voluntarily participate in this study and I am at least 18 years old.

End of Block: Informed Consent

Start of Block: Informed Consent (1)

**This survey asks questions about your experiences during the COVID-19 pandemic. This is a historic time. Your participation in this project is very important.**
  
 **Your answers are anonymous. No one will be able to link your answers to you. We will pay $1.50 for completing the survey which takes approximately 25 minutes.**
  
 **We will be asking you to complete a follow-up survey again in three months. We will contact you through MTurk when it is time to complete the follow-up survey.

 Any questions about the research or your participation in the research should be directed to the Principal Investigator, Dr. William O'Brien at wobrien@bgsu.edu or 419-372-2974. Alternatively, you may also contact the Chair of the Bowling Green State University Institutional Review Board, at 419-372-7716 or irb@bgsu.edu.**
  
 **Thank You! The survey Questions will now begin.**

End of Block: Informed Consent (1)

Start of Block: ID

Please create and enter a 4-character code you will use for the follow-up survey. To create the code, please enter the first two letters of your first name and the last two digits of your high school graduation year (e.g., William, 2010 = wi10). 

________________________________________________________________

End of Block: ID

Start of Block: Captcha

Before you proceed to the survey, please complete the captcha below.

End of Block: Captcha

Start of Block: PHQ-15

During the past month, how much have you been bothered by any of the following problems:

|  | Not bothered at all | Bothered a little | Bothered a lot |
| --- | --- | --- | --- |
| Stomach pain |  |  |  |
| Back pain |  |  |  |
| Pain in your arms or legs or other joints |  |  |  |
| Headaches |  |  |  |
| Chest Pain |  |  |  |
| Dizziness |  |  |  |
| Fainting spells |  |  |  |
| Feeling your heart pound or race |  |  |  |
| Shortness of breath |  |  |  |
| Pain or problems during sexual intercourse |  |  |  |
| Constipation, loose bowels, or diarrhea |  |  |  |
| Nausea, gas, or indigestion |  |  |  |
| Feeling tired, or having low energy |  |  |  |
| Trouble sleeping |  |  |  |

End of Block: PHQ-15

Start of Block: Vaccine Status/VHS

Have you received a COVID-19 vaccination?

- Yes
- No

| Page Break |  |
| --- | --- |

Which vaccine did you receive?

- Pfizer
- Moderna
- Johnson and Johnson
- AstraZeneca
- Sinovac
- Baharat
- Novavax
- Sputnik
- Other (please write in text box) ________________________________________________

When did you receive the vaccine?

- Month ________________________________________________
- Year ________________________________________________

| Page Break |  |
| --- | --- |

Please rate the extent to which you agree with each of the statements below.

|  | Strongly Disagree | Disagree | Neither Agree or disagree | Agree | Strongly agree |
| --- | --- | --- | --- | --- | --- |
| I am completely confident that COVID-19 vaccines are safe. |  |  |  |  |  |
| COVID-19 vaccines are effective. |  |  |  |  |  |
| Regarding COVID-19 vaccines, I am confident that public authorities decided in the best interests of the community. |  |  |  |  |  |
| A COVID-19 vaccination is unnecessary because COVID-19 is not common. |  |  |  |  |  |
| My immune system is strong. Without a vaccine it would have protected me against COVID-19. |  |  |  |  |  |
| COVID-19 is not so severe, so I do not need to get vaccinated. |  |  |  |  |  |
| Every day stress made it difficult to get the COVID-19 vaccine. |  |  |  |  |  |
| For me, it was inconvenient to receive the COVID-19 vaccine. |  |  |  |  |  |
| Visiting the doctor made me feel uncomfortable; this made it hard to get a COVID-19 vaccine. |  |  |  |  |  |
| Before getting the COVID-19 vaccination, I weighed the benefits and risks to make the best decision possible. |  |  |  |  |  |
| For the COVID-19 vaccination, I closely considered whether it is useful for me or not. |  |  |  |  |  |
| It was important for me to fully understand the topic of COVID-19 vaccination before I got vaccinated. |  |  |  |  |  |
| When everyone else has gotten a COVID-19 vaccination, others will not need to get vaccinated. |  |  |  |  |  |
| Getting a COVID-19 vaccine can also protect people with a weaker immune system. |  |  |  |  |  |
| COVID-19 vaccination is a collective action to prevent the spread of COVID-19. |  |  |  |  |  |
| I feel less anxiety and worry since I received the vaccine. |  |  |  |  |  |

| Page Break |  |
| --- | --- |

Please rate the extent to which you agree with each of the statements below.

|  | Strongly disagree | Disagree | Neither agree or disagree | Agree | Strongly agree |
| --- | --- | --- | --- | --- | --- |
| I will get a COVID-19 vaccine when it becomes available to me. |  |  |  |  |  |
| I will wait until a future date to decide whether I will get a COVID-19 vaccine. |  |  |  |  |  |
| I will search for information on where, when and how I can get vaccinated against COVID-19. |  |  |  |  |  |
| I will make an appointment at a medical facility in order to get vaccinated against COVID-19. |  |  |  |  |  |
| I will vaccinated myself against COVID-19. |  |  |  |  |  |

Please rate the extent to which you agree with each of the statements below.

|  | Strongly disagree | Disagree | Neither agree nor disagree | Agree | Strongly agree |
| --- | --- | --- | --- | --- | --- |
| I am completely confident that COVID-19 vaccines are safe. |  |  |  |  |  |
| COVID-19 vaccines are effective. |  |  |  |  |  |
| Regarding COVID-19 vaccines, I am confident that public authorities decided in the best interests of the community. |  |  |  |  |  |
| A COVID-19 vaccination is unnecessary because COVID-19 is not common. |  |  |  |  |  |
| My immune system is strong. It also will protect me against COVID-19. |  |  |  |  |  |
| COVID-19 is not so severe, so I do not need to get vaccinated. |  |  |  |  |  |
| Every day stress has prevented me from getting a COVID-19 vaccine. |  |  |  |  |  |
| For me, it is inconvenient to receive the COVID-19 vaccine. |  |  |  |  |  |
| Visiting a doctor makes me feel uncomfortable; this keeps me from getting a COVID-19 vaccine. |  |  |  |  |  |
| When I think about getting a COVID-19 vaccine, I have weighed the benefits and risks to make the best decision possible. |  |  |  |  |  |
| For the COVID-19 vaccination, I closely consider whether it is useful for me or not. |  |  |  |  |  |
| It is important for me to fully understand the topic of COVID-19 vaccination before I get vaccinated. |  |  |  |  |  |
| When everyone else has gotten a COVID-19 vaccination, I will not need to get vaccinated. |  |  |  |  |  |
| Getting a COVID-19 vaccine can also protect people with a weaker immune system. |  |  |  |  |  |
| COVID-19 vaccination is a collective action to prevent the spread of COVID-19. |  |  |  |  |  |
| I feel more anxious and worried because I have not yet received the vaccine. |  |  |  |  |  |

| Page Break |  |
| --- | --- |

Please rate the extent to which you agree with each of the statements below.

|  | Strongly disagree | Disagree | Neither agree nor disagree | Agree | Strongly agree |
| --- | --- | --- | --- | --- | --- |
| Getting vaccinated for COVID-19 seems to me to be Very Unwise. |  |  |  |  |  |
| Getting vaccinated for COVID-19 seems to me to be Very Wise. |  |  |  |  |  |
| Getting vaccinated for COVID-19 seems to me to be Unprotective. |  |  |  |  |  |
| Getting vaccinated for COVID-19 seems to me to be Protective. |  |  |  |  |  |
| Getting vaccinated for COVID-19 seems to me to be Very Harmful. |  |  |  |  |  |
| Getting vaccinated for COVID-19 seems to me to be Very Beneficial. |  |  |  |  |  |
| Most people like me will get vaccinated for COVID-19. |  |  |  |  |  |
| Most people who are important to me will get vaccinated for COVID-19. |  |  |  |  |  |
| Most people who are important to me think I should get vaccinated for COVID-19. |  |  |  |  |  |
| If I wanted to, I could go to a medical facility with no problems whatsoever and get vaccinated. |  |  |  |  |  |
| Getting vaccinated against COVID-19 would be very hard for me. |  |  |  |  |  |
| Getting vaccinated for COVID-19 would be very easy for me. |  |  |  |  |  |
| I have full control about when and where I get the COVID-19 vaccine. |  |  |  |  |  |
| I have no control at all about when and where I get the COVID-19 vaccine. |  |  |  |  |  |
| COVID-19 vaccines are effective in preventing me from getting the illness. |  |  |  |  |  |
| COVID-19 vaccines are effective in preventing me from passing the illness to others. |  |  |  |  |  |
| I trust the information I receive from medical sources about COVID-19. |  |  |  |  |  |
| I trust the information I receive from the government about COVID-19. |  |  |  |  |  |
| I trust the information I receive from social networks (e.g. Facebook, Twitter, Line, WeChat) about COVID-19. |  |  |  |  |  |

End of Block: Vaccine Status/VHS

Start of Block: QOL

This assessment asks how you feel about your quality of life, health, or other areas of your life. If you are unsure about which response to give to a question, please choose the one that appears most appropriate. We ask that you think about your life in the last month.

How would you rate your quality of life?

- very poor
- poor
- Neither poor nor good
- good
- very good

How satisfied are you with your health?

- very dissatisfied
- dissatisfied
- neither satisfied nor disatisfied
- satisfied
- very satisfied

How satisfied are you with your capacity for work?

- very dissatisfied
- dissatisfied
- neither satisfied nor disatisfied
- satisfied
- very satisfied

How much do you enjoy life?

- very little
- little
- neutral
- much
- very much

To what extent do you feel your life to be meaningful?

- very little
- little
- neutral
- much
- very much

Have you enough money to meet your needs?

- not at all
- a little
- moderately
- mostly
- completely

| Page Break |  |
| --- | --- |

Indicate for each of the statements below the degree to which this change occurred in your life **as a result of the COVID-19 pandemic.**

|  | I did not experience this change | I experienced this change to a very small degree | I experienced this change to a small degree | I experienced this change to a moderate degree | I experienced this change to a great degree | I experienced this change to a very great degree |
| --- | --- | --- | --- | --- | --- | --- |
| I changed my priorities about what is important in life. |  |  |  |  |  |  |
| I have a greater appreciation for the value of my own life. |  |  |  |  |  |  |
| I have a better understanding of spiritual matters. |  |  |  |  |  |  |
| I established a new path for my life. |  |  |  |  |  |  |
| I have a greater sense of closeness with others. |  |  |  |  |  |  |
| I know better that I can handle difficulties. |  |  |  |  |  |  |
| I am able to do better things with my life. |  |  |  |  |  |  |
| I have stronger religious faith. |  |  |  |  |  |  |
| I discovered that I am stronger than I thought I was. |  |  |  |  |  |  |
| I learned a great deal about how wonderful people are. |  |  |  |  |  |  |

End of Block: QOL

Start of Block: GHQ

We would like to know how your health has been in general, over the past month. Please select the answer that best applies to your experience of the last month.

|  | Much less than usual | Less than usual | Same as usual | More than usual | Much more than usual |
| --- | --- | --- | --- | --- | --- |
| Been able to concentrate on whatever you are doing? |  |  |  |  |  |
| Lost sleep over worry? |  |  |  |  |  |
| Felt that you were playing a useful part in things? |  |  |  |  |  |
| Felt capable of making decisions about things? |  |  |  |  |  |
| Felt constantly under strain? |  |  |  |  |  |
| Felt that you couldn't overcome your difficulties? |  |  |  |  |  |
| Been able to enjoy your normal day-to-day activities? |  |  |  |  |  |
| Been able to face up to your problems? |  |  |  |  |  |
| Been feeling unhappy and depressed? |  |  |  |  |  |
| Been losing self-confidence in yourself? |  |  |  |  |  |
| Been thinking of yourself as a worthless person? |  |  |  |  |  |
| Been feeling reasonably happy, all things considered? |  |  |  |  |  |

Please answer the captcha below to proceed with the survey.

How likely is it that you will contract the Coronavirus (COVID-19)? Please base your estimate on the following rating scale?

- No chance
- Unlikely
- About equally likely to happen or not happen
- Likely
- Certain

End of Block: GHQ

Start of Block: PVD

Please rate the extent to which you agree with each of the statements below.

|  | Strongly disagree | Disagree | Neutral | Agree | Strongly agree |
| --- | --- | --- | --- | --- | --- |
| If an illness is 'going around', I will get it. |  |  |  |  |  |
| My past experiences make me believe I am not likely to get sick even when my friends are sick. |  |  |  |  |  |
| I have a history of susceptibility to infectious diseases. |  |  |  |  |  |
| In general, I am very susceptible to colds, flu, and other infectious diseases. |  |  |  |  |  |
| I am more likely than the people around me to catch an infectious disease. |  |  |  |  |  |
| My hands do not feel dirty after touching money. |  |  |  |  |  |
| I am unlikely to catch a cold, flu, or other illness, even if it is going around. |  |  |  |  |  |
| My immune system protects me from most illnesses that other people get. |  |  |  |  |  |
| Please select 'Disagree' for this question |  |  |  |  |  |

End of Block: PVD

Start of Block: IUS-SF

Please rate how much you agree with each item:

|  | Not at all characteristic of me | A little characteristic of me | Somewhat characteristic of me | Very characteristic of me | Entirely characteristic of me |
| --- | --- | --- | --- | --- | --- |
| Unforeseen events upset me greatly. |  |  |  |  |  |
| It frustrates me not having all the information I need. |  |  |  |  |  |
| Uncertainty keeps me from living a full life. |  |  |  |  |  |
| One should always look ahead so as to avoid surprises. |  |  |  |  |  |
| A small unforeseen event can spoil everything, even with the best of planning. |  |  |  |  |  |
| When it's time to act, uncertainty paralyses me. |  |  |  |  |  |
| When I am uncertain I can't function very well. |  |  |  |  |  |
| I always want to know what the future has in store for me. |  |  |  |  |  |
| I can't stand being taken by surprise. |  |  |  |  |  |
| The smallest doubt can stop me from acting. |  |  |  |  |  |
| I should be able to organize everything in advance. |  |  |  |  |  |
| I must get away from all uncertain situations. |  |  |  |  |  |

End of Block: IUS-SF

Start of Block: PATS

Please read each statement and rate how much the statement applies to you. There are no right or wrong answers. Do not spend too much time on any statement.

|  | Does not apply to me at all | Applies to me to some degree, or some of the time | Applies to me to a considerable degree or a good part of time | Applies to me very much or most of the time |
| --- | --- | --- | --- | --- |
| I avoid public events and crowded places. |  |  |  |  |
| I avoid physical contact with other people. |  |  |  |  |
| I wear a face mask outside of my home. |  |  |  |  |
| I wear gloves outside of my home. |  |  |  |  |
| Please answer "applies to me very much or most of the time" for this item |  |  |  |  |

End of Block: PATS

Start of Block: IOES-R

The following is a list of difficulties people sometimes have after stressful life events. Please read each item, and then indicate how distressing each difficulty has been for you ***during the past 7 days*** with respect to COVID-19. How much were you distressed or bothered by these difficulties?

|  | Not at all | A little bit | Moderately | Quite a bit | Extremely |
| --- | --- | --- | --- | --- | --- |
| Any reminder brought back feelings about it. |  |  |  |  |  |
| I had trouble staying asleep. |  |  |  |  |  |
| Other things kept making me think about it. |  |  |  |  |  |
| I felt irritable and angry. |  |  |  |  |  |
| I avoided letting myself get upset when I thought about it or was reminded of it. |  |  |  |  |  |
| I thought about it when I didn't mean to. |  |  |  |  |  |
| I felt as if it hadn't happened or wasn't real |  |  |  |  |  |
| I stayed away from reminders about it. |  |  |  |  |  |
| Pictures about it popped into my mind. |  |  |  |  |  |
| I was jumpy and easily startled. |  |  |  |  |  |
| I tried not to think about it. |  |  |  |  |  |
| I was aware that I still had a lot of feelings about it, but I didn't deal with them. |  |  |  |  |  |
| My feelings about it were kind of numb. |  |  |  |  |  |
| I found myself acting or feeling like I was back at that time. |  |  |  |  |  |
| I had trouble falling asleep. |  |  |  |  |  |
| I had waves of strong feelings about it. |  |  |  |  |  |
| I tried to remove it from my memory. |  |  |  |  |  |
| I had trouble concentrating. |  |  |  |  |  |
| Please select 'Moderately' for this item |  |  |  |  |  |
| Reminders of it caused me to have physical reactions, such as seating, trouble breathing, nausea, or a pounding heart. |  |  |  |  |  |
| I had dreams about it. |  |  |  |  |  |
| I felt watchful and on guard. |  |  |  |  |  |
| I tried not to talk about it. |  |  |  |  |  |

End of Block: IOES-R

Start of Block: The Compassion Scale

Instructions: Please read each statement carefully before answering. Please answer according to what really reflects your experience rather than what you think your experience should be. Indicate how often you behave in the stated manner, using the following scale:

|  | Almost Never | Sometimes | About half the time | Most of the time | Almost Always |
| --- | --- | --- | --- | --- | --- |
| I pay careful attention when other people talk to me about their troubles. |  |  |  |  |  |
| I am unconcerned with other people’s problems. |  |  |  |  |  |
| I realize everyone feels down sometimes, it is part of being human. |  |  |  |  |  |
| I notice when people are upset, even if they don’t say anything. |  |  |  |  |  |
| I like to be there for others in times of difficulty. |  |  |  |  |  |
| I feel it’s important to recognize that all people have weaknesses and no one’s perfect. |  |  |  |  |  |
| I listen patiently when people tell me their problems. |  |  |  |  |  |
| My heart goes out to people who are unhappy. |  |  |  |  |  |
| I try to avoid people who are experiencing a lot of pain. |  |  |  |  |  |
| I feel that suffering is just a part of the common human experience. |  |  |  |  |  |
| When people tell me about their problems, I try to keep a balanced perspective on the situation. |  |  |  |  |  |
| When others feel sadness, I try to comfort them. |  |  |  |  |  |
| I can’t really connect with other people when they’re suffering. |  |  |  |  |  |
| Despite my differences with others, I know that everyone feels pain just like me. |  |  |  |  |  |

End of Block: The Compassion Scale

Start of Block: The Individualism and Collectivism Scale/Culture Orientation Scale

All items are answered on a 5-point scale, ranging from 1= never or definitely no and 5 = always or definitely yes. Please respond to each of the following statements using the five point scale

|  | Never/Definitely No  1 | 2 | 3 | 4 | Always/Definitely Yes  5 |
| --- | --- | --- | --- | --- | --- |
| I'd rather depend on myself than others |  |  |  |  |  |
| I rely on myself most of the time; I rarely rely on others. |  |  |  |  |  |
| I often do "my own thing." |  |  |  |  |  |
| My personal identity, independent of others, is very important to me. |  |  |  |  |  |
| It is important that I do my job better than others. |  |  |  |  |  |
| Winning is everything. |  |  |  |  |  |
| Competition is the law of nature. |  |  |  |  |  |
| When another person does better than I do, I get tense and aroused. |  |  |  |  |  |
| If a coworker gets a prize, I would feel proud. |  |  |  |  |  |
| The well-being of my coworkers is important to me. |  |  |  |  |  |
| To me, pleasure is spending time with others. |  |  |  |  |  |
| I feel good when I cooperate with others. |  |  |  |  |  |
| Parents and children must stay together as much as possible. |  |  |  |  |  |
| It is my duty to take care of my family, even when I have to sacrifice what I want. |  |  |  |  |  |
| Family members should stick together, no matter what sacrifices are required. |  |  |  |  |  |
| It is important to me that I respect the decisions made by my groups. |  |  |  |  |  |

End of Block: The Individualism and Collectivism Scale/Culture Orientation Scale

Start of Block: Serenity Scale

All items are answered on a 5-point scale, ranging from 1= never and 5 = always. Please respond to each of the following statements using the five point scale

|  | Never  1 | 2 | 3 | 4 | Always  5 |
| --- | --- | --- | --- | --- | --- |
| I am forgiving of myself for past mistakes |  |  |  |  |  |
| I take care of today and let yesterday and tomorrow take care of themselves |  |  |  |  |  |
| In problem situations, I do what I am able to do and then accept whatever happens even if I dislike it |  |  |  |  |  |
| I accept situations that I cannot change |  |  |  |  |  |
| I try to place my problems in the proper perspective in any given situation |  |  |  |  |  |
| I find ways to share my talents with others |  |  |  |  |  |
| I attempt to deal with what is, rather than what was, or what will be |  |  |  |  |  |
| I feel that I have done the best I could in life |  |  |  |  |  |
| I feel forgiving of those who have harmed me |  |  |  |  |  |
| I feel serene |  |  |  |  |  |
| I am aware of an inner source of comfort, strength, and security |  |  |  |  |  |
| During troubled time, I experience an inner source of strength |  |  |  |  |  |
| I experience peace of mind |  |  |  |  |  |
| I am aware of inner peace |  |  |  |  |  |
| I experience an inner quiet that does not depend on events |  |  |  |  |  |
| When I get upset, I become peaceful by getting in touch with my inner self |  |  |  |  |  |
| I experience an inner calm even when I am under pressure |  |  |  |  |  |
| I can feel angry and observe my feeling of anger and separate myself from it and still feel an inner peace |  |  |  |  |  |
| Please select 'Always' for this item |  |  |  |  |  |
| I trust that life events happen to fit a plan which is larger and more gentle than I can know |  |  |  |  |  |
| I see the good in painful events that have happened to me |  |  |  |  |  |
| Even though I do not understand, I trust in the ultimate goodness of the plan of things |  |  |  |  |  |
| I trust that everything happens as it should |  |  |  |  |  |

End of Block: Serenity Scale

Start of Block: Authoritarianism Scale

Please respond to each of the following statements using the five point scale

|  | Strongly disagree  1 | 2 | 3 | 4 | Strongly agree  5 |
| --- | --- | --- | --- | --- | --- |
| It’s great that many young people today are prepared to defy authority. |  |  |  |  |  |
| What our country needs most is discipline, with everyone following our leaders in unity |  |  |  |  |  |
| God’s laws about abortion, pornography, and marriage must be strictly followed before it is too late |  |  |  |  |  |
| There is nothing wrong with premarital sexual intercourse. |  |  |  |  |  |
| Our society does NOT need tougher government and stricter laws. |  |  |  |  |  |
| The facts on crime and the recent public disorders show we have to crack down harder on troublemakers, if we are going preserve law and order. |  |  |  |  |  |

End of Block: Authoritarianism Scale

Start of Block: Mindful Insight Scale

The Mindful Insight Scale is in development. To obtain items, please write to Dr. Somboon Jarukasemthawee at Somboon.kla@gmail.com.

End of Block: Mindful Insight Scale

Start of Block: Medical Care Delays

Please respond to each of the following statements using the five point scale

|  | Strongly disagree  1 | 2 | 3 | 4 | Strongly agree  5 |
| --- | --- | --- | --- | --- | --- |
| In order to receive medical care during the COVID-19 pandemic, I had to choose between the risk of becoming infected with COVID-19 and the risk of my symptoms getting worse. |  |  |  |  |  |
| I'm worried that if I can not get enough medical treatment during COVID-19, my illnesses will get worse. |  |  |  |  |  |
| As a result of the COVID-19 outbreak, I've lost the opportunity to be as healthy as I would like to be |  |  |  |  |  |
| I have a health problem for which I was unable to receive medical treatment because of COVID-19. |  |  |  |  |  |

End of Block: Medical Care Delays

Start of Block: MPFI

Please rate the extent to which you agree with each of the statements below.
In the last 2 weeks,

|  | Never True | Rarely True | Occasionally True | Often True | Very Often True | Always True |
| --- | --- | --- | --- | --- | --- | --- |
| I was receptive to observing unpleasant thoughts and feelings without interfering with them |  |  |  |  |  |  |
| I tried to make peace with my negative thoughts and feelings rather than resisting them |  |  |  |  |  |  |
| I was attentive and aware of my emotions |  |  |  |  |  |  |
| I was in tune with my thoughts and feelings from moment to moment |  |  |  |  |  |  |
| Even when I felt hurt or upset, I tried to maintain a broader perspective |  |  |  |  |  |  |
| I carried myself through tough moments by seeing my life from a larger viewpoint |  |  |  |  |  |  |
| I was able to let negative feelings come and go without getting caught up in them |  |  |  |  |  |  |
| When I was upset, I was able to let those negative feelings pass through me without clinging to them |  |  |  |  |  |  |
| I was very in-touch with what is important to me and my life |  |  |  |  |  |  |
| I stuck to my deeper priorities in life |  |  |  |  |  |  |
| Even when I stumbled in my efforts, I didn't quit working toward what is important |  |  |  |  |  |  |
| Even when times got tough, I was still able to take steps toward what I value in life |  |  |  |  |  |  |
| When I had a bad memory, I tried to distract myself to make it go away |  |  |  |  |  |  |
| I tried to distract myself when I felt unpleasant emotions |  |  |  |  |  |  |
| I did most things on "automatic" with little awareness of what I was doing |  |  |  |  |  |  |
| I did most things mindlessly without paying much attention |  |  |  |  |  |  |
| I thought some of my emotions were bad or inappropriate and I shouldn't feel them |  |  |  |  |  |  |
| I criticized myself for having irrational or inappropriate emotions |  |  |  |  |  |  |
| Negative thoughts and feelings tended to stick with me for a long time. |  |  |  |  |  |  |
| Distressing thoughts tended to spin around in my mind like a broken record. |  |  |  |  |  |  |
| My priorities and values often fell by the wayside in my day to day life |  |  |  |  |  |  |
| When life got hectic, I often lost touch with the things I value |  |  |  |  |  |  |
| Negative feelings often trapped me in inaction |  |  |  |  |  |  |
| Negative feelings easily stalled out my plans |  |  |  |  |  |  |

End of Block: MPFI

Start of Block: SCS

Please read each statement carefully before answering. To the right of each item, indicate how often you behave in the stated manner, using the following scale:

|  | Almost Never | Sometimes | About half the time | Most of the time | Almost Always |
| --- | --- | --- | --- | --- | --- |
| When I fail at something important to me I become consumed by feelings of inadequacy. |  |  |  |  |  |
| I try to be understanding and patient towards those aspects of my personality I don’t like. |  |  |  |  |  |
| When something painful happens I try to take a balanced view of the situation. |  |  |  |  |  |
| When I’m feeling down, I tend to feel like most other people are probably happier than I am. |  |  |  |  |  |
| I try to see my failings as part of the human condition. |  |  |  |  |  |
| When I’m going through a very hard time, I give myself the caring and tenderness I need. |  |  |  |  |  |
| When something upsets me I try to keep my emotions in balance. |  |  |  |  |  |
| When I fail at something that’s important to me, I tend to feel alone in my failure |  |  |  |  |  |
| When I’m feeling down I tend to obsess and fixate on everything that’s wrong. |  |  |  |  |  |
| When I feel inadequate in some way, I try to remind myself that feelings of inadequacy are shared by most people. |  |  |  |  |  |
| I’m disapproving and judgmental about my own flaws and inadequacies. |  |  |  |  |  |
| I’m intolerant and impatient towards those aspects of my personality I don’t like. |  |  |  |  |  |

End of Block: SCS

Start of Block: Pandemic Fatigue Scale

Please rate the extent to which you agree with each of the statements below.

|  | Strongly disagree | Disagree | Somewhat disagree | Neither agree nor disagree | Somewhat agree | Agree | Strongly agree |
| --- | --- | --- | --- | --- | --- | --- | --- |
| It bothers me to adhere to the behavioral guidelines. |  |  |  |  |  |  |  |
| I am tired of restricting my liberty to avoid the spread of COVID-19. |  |  |  |  |  |  |  |
| I am tired of all the COVID-19 discussions in TV shows, newspapers, and radio programs, etc. |  |  |  |  |  |  |  |
| I am exhausted from trying to keep up with the constantly changing recommendations around COVID-19. |  |  |  |  |  |  |  |
| I feel strained from following all of the behavioral regulations and recommendations around COVID-19. |  |  |  |  |  |  |  |
| I am sick of hearing about COVID-19. |  |  |  |  |  |  |  |
| I am tired of restraining myself to save those who are most vulnerable to COVID-19. |  |  |  |  |  |  |  |
| These days I am finding it more and more difficult to force myself to follow the COVID-19 regulations. |  |  |  |  |  |  |  |
| When friends or family members talk about COVID-19, I try to change the subject because I do not want to talk about it anymore. |  |  |  |  |  |  |  |
| I am losing my spirit to fight against COVID-19. |  |  |  |  |  |  |  |

End of Block: Pandemic Fatigue Scale

Start of Block: Demographics (updated 7/2021)

Please enter your age (in years)?

________________________________________________________________

Which of these best describes your sex assigned at birth?

- Male
- Female
- Intersex ________________________________________________

Which of these best describes your current gender identify (please check all that apply)?

- Female/woman
- Male/man
- Gender nonbinary/gender fluid
- Transgender female/woman
- Transgender male/man
- Other not listed: (Please specify) ________________________________________________

Which of these best describes your sexual orientation (please check all that apply)?

- Asexual
- Bisexual
- Gay/Lesbian
- Heterosexual/Straight
- Pansexual
- Queer
- Other not listed: (Please specify) ________________________________________________

What is your occupation?

________________________________________________________________

Which of the following best describes your CURRENT employment arrangement?

- Employed, working 1-24 hours per week
- Employed, working 24-39 hours per week
- Employed, working 40 or more hours per week
- Not employed, looking for work
- Not employed, NOT looking for work
- Retired
- Disabled, not able to work

What is your marital status?

- Single
- Cohabiting (not married)
- Long term relationship (not married or cohabiting)
- Married
- Divorced
- Widowed
- Other (please specify) ________________________________________________

How many dependent children do you have?

________________________________________________________________

End of Block: Demographics (updated 7/2021)

Start of Block: Demographics3

Please indicate your pretax annual income in dollars.

- 0 - 10,000
- 10,001 - 20,000
- 20,001 - 30,000
- 30,001 - 40,000
- 40,001 - 50,000
- 50,001 - 60,000
- 60,001 - 70,000
- 70,001 - 80,000
- 80,001 - 90,000
- 90,001 - 100,000
- 100,001 - 120,000
- 120,001 - 140,000
- 140,001 - 160,000
- 160,001 - 180,000
- 180,001 - 200,000
- 200,001 +

Please select "About half of the time" for this item.

- Never
- Sometimes
- About half of the time
- Most of the time
- Always

| 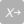 |
| --- |

Do you have any medical conditions (not coronavirus)?

- Yes, please list ________________________________________________
- No

| 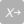 |
| --- |

Are you currently taking any medicine?

- Yes, please list ________________________________________________
- No

What is your height?

- Feet ________________________________________________
- Inches ________________________________________________

What is your weight in pounds?

________________________________________________________________

End of Block: Demographics3

Start of Block: Demographics4

Please enter your age?

________________________________________________________________

What is your occupation?

________________________________________________________________

Do you have children?

- Yes
- No

Regarding Coronavirus (COVID-19):

- I am not infected, but I have not been tested
- I am not infected, based on negative results from a COVID-19 antibody test or nasal swab
- I could be currently infected, but I have not been tested
- I am currently infected based on a positive COVID-19 test result
- I was not infected in the past, but I have not been tested
- I could have been infected in the past, but I have not been tested
- I was infected in the past based on positive results from a COVID-19 antibody test or nasal swab

What is your racial and cultural identity? Please choose the race/ethnicity category with which you primarily identify.

- Hispanic or Latino: a person of Cuban, Mexican, Chicano, Puerto Rican, South or Central American, or other Spanish culture or origin, regardless of race. Please write in the origin of your identity (e.g., Cuban, Mexican, Salvadoran, Columbian, etc.)
- White: a person having origins in any of the original peoples of Europe, the Middle East, or North Africa. Please write in origin of your identity (e.g., Irish, German, Italian, English, French, Ect.)
- Black or African American: a person having origins in any of the black racial groups of Africa. Please write in origin of your identity (e.g., African American, Jamaican, Nigerian, Ethiopian, ect.)
- Asian: a person having origins in any of the original peoples of the Far East, Southeast Asia, or the Indian subcontinent including, for example, Cambodia, China, India, Japan, Korea, Malaysia, Pakistan, the Philippine Islands, Thailand, and Vietnam. Please write in origin of your identity (e.g., China, Cambodia, Thailand, Vietnam, Korea, etc.)
- Native Hawaiian or Other Pacific Islander: a person having origins in any of the original peoples of North and South America (including Central America), and who maintains tribal affiliation or community attachment. Please write in origin of your identity (e.g., Native Hawaiian, Fijian, Filipino, ect.)
- American Indian or Alaska Native: a person having origins in any of the original peoples of North and South America (including Central America), and who maintains tribal affiliation or community attachment. Please write in origin of your identity (e.g., Navajo Nation, Mayan, Aztec, Nome Eskimo, etc.)
- Two or more Races: a person who primarily identifies with two or more of the above race/ethnicity categories.
- I choose not to self-identify my race/ethnicity at this time

In what country are you currently living?

________________________________________________________________

In what state are you currently living?

________________________________________________________________

In what city are you currently living?

________________________________________________________________

What type of accommodation are you currently living?

- Own home/apartment
- Parent's home/apartment
- Friend's home/apartment
- Hotel
- University accommodation
- Other temporary home/apartments
- Hospital
- Nursing home/Assisted living
- Quarantine
- Other (please specify)

| 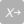 |
| --- |

Are you currently in self-isolation?

- Yes
- No

How many times do you leave your place in a typical day?

________________________________________________________________

End of Block: Demographics4
